# American Public Health Association

**Published:** 1896-09

**Authors:** Byron H. Daggett

**Affiliations:** Chairman of Committee on Exhibits; 258 Franklin St.; Buffalo, N. Y.


### American Public Health Association.

BUFFALO, N. Y., July 20, 1896.

*Gentlemen:*

The local committee appointed for the purpose of entertaining the American Public Health Association regards it desirable to provide for an exhibition of sanitary goods and appliances as a source of instruction and usefulness to the public, as well as of benefit to those engaged in the manufacture and sale of such material, including all other ways and means of promoting health.

In order to provide for the unavoidable expenses incurred for this purpose, each exhibitor is to be charged for the amount of floor area occupied by himself.

The charge for space will be 40 cents per square foot.

The choice of location will be allotted in the order of the applications, first come first served. Ten per cent of the amount charged is to be paid when the space is assigned, to secure its reservation. The balance to fall due on the opening of the meeting, September 15.

The Ellicott Square Building is one of the most complete business structures in America, and it is located in the heart of the great commercial city of Buffalo.

Those dealing in goods pertaining to health and sanitation can not find a better mart for their wares or a better opportunity for displaying them. That there may be no confusion or misunderstanding, let me suggest that you engage space promptly, so that it may be reserved. Possession may be had during the meeting or at any time during the prior fortnight.

We address you this invitation, hoping that you will share our enthusiasm and join us on that auspicious occasion.

Very respectfully,

BYRON H. DAGGETT, M. D.,

258 Franklin St. Chairman of Committee on Exhibits.

